# Refractive outcomes for secondary sutureless posterior chamber lens implantation: sutureless scleral fixating lens Carlevale® versus retropupillary iris-claw lens Artisan®

**DOI:** 10.1007/s00417-024-06683-8

**Published:** 2024-11-11

**Authors:** Justine Bontemps, Olivier Loria, Lucas Sejournet, Benoit Allignet, Sandra Elbany, Frédéric Matonti, Carole Burillon, Philippe Denis, Laurent Kodjikian, Thibaud Mathis

**Affiliations:** 1 Service d’Ophtalmologie, Hôpital de La Croix-Rousse, Hospices Civils de Lyon, 69004 Lyon, France; 2 Université Claude Bernard Lyon 1, 69100 Villeurbane, France; 3 Laboratoire MATEIS, UMR-CNRS 5510, INSA, Université Lyon 1, 69100 Villeurbanne, France; 4 Service de Radiothérapie, Centre Léon Bérard, 28 Rue Laennec, 69673 Lyon, France; 5 Laboratoire CREATIS, CNRS UMR 5220, Inserm U1206, INSA-Lyon, Université Jean Monnet Saint-Étienne, Université Claude Bernard Lyon1, 69621 Villeurbanne, France; 6 Service d’Ophtalmologie, Hôpital Edouard Herriot, Hospices Civils de Lyon, 69003 Lyon, France; 7 Centre Monticelli Paradis Center, 13000 Marseille, France; 8 Institut de Neurosciences de La Timone, Université d’Aix-Marseille, 13000 Marseille, France

**Keywords:** Aphakia, Artisan®, Carlevale®, Iris-claw implant, Scleral-fixated sutureless intraocular lens, Secondary implantation, Surgically induced astigmatism

## Abstract

**Purpose:**

To compare refractive outcomes of the foldable intraocular lens sutureless scleral fixated sutureless (Carlevale® FIL-SSF) with the iris-claw lens (Artisan®).

**Methods:**

This retrospective study included consecutive patients who underwent a FIL-SSF implantation or an iris-claw implantation between January 2020 and November 2022 in the ophthalmology departments of Hospices Civils de Lyon (France).

**Results:**

A total of 271 eyes from 265 patients were included: 96 eyes in the FIL-SSF group and 175 eyes in the iris-claw group. At 6 months, the mean (SD) surgically induced astigmatism (SIA) was significantly lower in the FIL-SSF group with 0.3 (1.8) diopters against 0.8 (2.1) diopters in the iris-claw group (*p* = 0.01). The mean (SD) refractive error was also lower for the FIL-SSF group with 0.1 (1.2) diopters versus 0.5 (1.6) diopters in the iris-claw group (*p* < 0.001). The mean best corrected visual acuity at 6 months was not significantly different between FIL-SSF and iris-claw lens with 0.47 (0.58) logMAR and 0.39 (0.55) logMAR, respectively (*p* = 0.12). However, the mean (SD) operative time was longer for FIL-SSF implantation in comparison to iris-claw implantation (59.8 (21.1) minutes versus 41.9 (24.4) minutes, respectively (*p* < 0.001)). The rate of postoperative complications was similar between the two techniques.

**Conclusion:**

This study shows that FIL-SSF achieves better refractive results than iris-claw lens, with a similar rate of postoperative complications. As a relatively new implantation technique, there is a learning curve required to reduce operating time.

**Key messages:**

***What is known?***

- Multiple surgical options for correcting aphakia in the absence of capsular support can be used.
- Currently, foldable intraocular lens sutureless scleral fixated sutureless (FIL-SSF, Carlevale®) and iris-claw (Artisan®) implants are the two preferred options, but there is no consensus on the best technique to adopt.

***What is new?***

- We showed that FIL-SSF has a significantly lower surgically induced astigmatism compared to the iris-claw implant.
- Similar rate of postoperative complications was found between these two techniques.
- Future studies with a longer follow-up period are needed to ascertain its tolerance.

## Introduction

Over the last decade, the surgical options for correcting aphakia in the absence of capsular support have evolved considerably, and management can now be customized according to the clinical situation [1]. Multiple surgical techniques are employed, such as placing the intraocular lens (IOL) in the anterior chamber [2], fixation to the posterior surface of the iris (with iris-claw or suture) [3–6] or fixation to the sclera (with or without suture) [7]. There are various surgical approaches described in the literature but there is no consensus on the best strategy to adopt [1]. The most commonly used technique is the retro-pupillary iris-claw implantation, introduced in 1978 [8] and popularized in 2002 [9], due to its ease of use. It offers a short learning curve, reduced operating time, and does not require a complete vitrectomy, making it available to all ophthalmic surgeons [10, 11]. However, the integrity of the iris diaphragm and the need for a large corneal incision which may induce postoperative astigmatism [12] are the two main limitations of this surgical technique [1].

In order to bypass the iris support deficiency, especially in cases of eye trauma, scleral fixated IOLs were introduced in 1981 [13], and since then, several variations have been proposed [14–17]. The latest is the FIL- SSF (foldable intraocular lens sutureless scleral fixated), which was developed by Carlevale and introduced in 2020 [18]. This foldable device can be implanted through a small incision of 2.2 mm (mm) and is anchored in the sclera through two sclerotomies. Several publications have since reported this technique as an effective and safe surgical strategy [19–21], but there is limited data on its refractive outcomes and only few, small sized studies comparing it with others surgical techniques [22, 23].

Therefore, the purpose of this study is to compare FIL-SSF with iris-claw lens, with a focus on the refractive outcomes and surgical induced astigmatism (SIA). We also compared visual outcomes and occurrence of peroperative and postoperative complications.

## Materials and methods

### Patients selection

We conducted a retrospective, multicenter study of all consecutive patients who underwent a FIL-SSF implantation or an iris-claw lens implantation between January 2020 and November 2022 in the ophthalmology departments of Croix Rousse and Edouard Herriot University Hospitals, Hospices Civils de Lyon, France. Patients were included if the follow-up data was at least 6 months after the surgery. Patients were excluded if they underwent penetrating keratoplasty (PKP) or descemet stripping automated endothelial keratoplasty (DSAEK) during the same surgery. Patients with corneal diseases that could influence astigmatism (keratoconus, corneal dystrophia…), were also excluded. An international review board approved the study (Ethics Committee of the French Society of Ophthalmology, IRB 00008855 Société Française d’Ophtalmologie IRB#1), with consideration to the tenets outlined in the Declaration of Helsinki.

### Data collection

The data on the study population were drawn from digital medical records. Patients were evaluated at baseline before the secondary implantation surgery, at 1 month and at 6 months after the surgery. Baseline data included demographic characteristics, comorbidities, best corrected visual acuity (BCVA), axial length, preoperative spherical equivalent (SE), astigmatism, intraocular pressure (IOP), indication for the surgery, and the IOL power with the expected spherical value as calculated from biometric data. Ocular hypotony was considered as IOP < 6 mmHg, and ocular hypertension was considered as IOP ≥ 25 mmHg. Each patient underwent slit lamp examination, a dilated fundus examination and an Optical Coherence Tomography exam (OCT SD, Heidelberg Engineering, Germany). Biometry was performed in all patients using the IOL Master 700 (Carl Zeiss, Oberkochen, Germany), and the IOL power was calculated with the SRK-T formula, in accordance with previous literature [19, 24]. Intraoperative data included the anesthesia type, the duration of the surgery, the use of a scleral tunnel or a corneal incision, and intraoperative complications. The surgeries were performed under general anesthesia, retrobulbar or subtenon injection, or sedation with topical anesthesia. The choice of anesthesia was determined by the surgeon, in agreement with the patient and the anesthesiologist. Combined surgeries included complete vitrectomy in all FIL-SSF patients and in iris-claw patients when necessary. In some cases, explantation of dislocated IOL by corneal or scleral incision was performed during the same surgery. One month and six months after the surgery, each patient underwent slit lamp examination, dilated fundus examination and OCT exam. Follow up data included BCVA, subjective refraction, IOP, and postoperative complications.

### Outcome measures

The primary outcome measure was the SIA at 6 months follow-up, defined as the difference between preoperative and postoperative astigmatism. The secondary outcome measures were mean refractive error (MRE) defined as the difference between subjective postoperative SE and the refraction predicted by the preoperative biometry, BCVA, duration of surgery and peroperative and postoperative complications. We also performed a subgroup analysis, comparing SIA in the FIL-SSF patients and in a subgroup of the iris-claw patients when a sclero-corneal tunnel was used instead of a direct corneal incision. Only subjective refraction was used to closely approximate the patient’s real-life visual quality. BCVA were expressed in a logMAR scale.

### IOL and surgical procedures

#### Carlevale® FIL-SSF

**Fig. 1:**
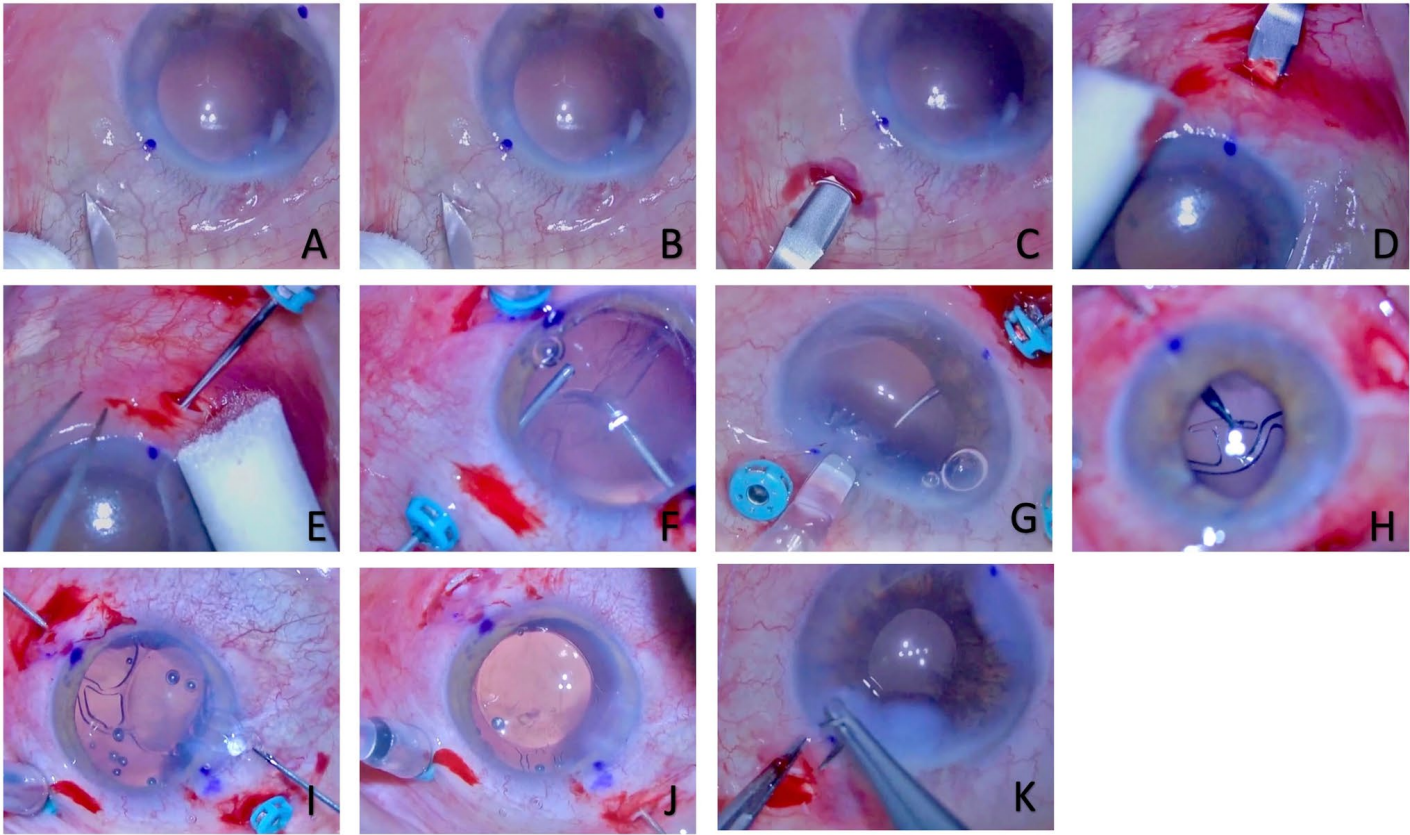
**A** Corneal marking along an axis at 180°, 2 mm from the limbus, followed by conjunctival peritomy. **B** Scleral incision 2 mm from the limbus. **C. D.** Creation of scleral pockets using a Crescent knife. **E** Realization of two 25G sclerotomies, within the incisions 2 mm from the limbus. **F** Cleaning of residual capsular bag debris if necessary. **G** Gentle and gradual injection of the implant into the anterior chamber through the 2.2 mm corneal incision. **H** The second plug is grasped using a crocodile grasping forceps. **I** Externalization of the haptic through the 25G sclerotomy. **J** The implant is automatically centered. **K** Hydrosuture of the corneal incision, followed by conjonctival sutures using Vicryl 8/0

The Carlevale® FIL-SSF is a uniquely designed, foldable, acrylic IOL with 25% H_2_O and UV filter. It has an optical diameter of 6.5 mm and a total diameter of 13.2 mm. It is equipped with a plug (2 mm × 1 mm) attached almost perpendicularly to the haptic’s long side. Prior to injection, one axial, or two radial scleral flaps were constructed at 180° from each other, and vitrectomy ports were placed within. A compass was used to position the anchors precisely 2 mm from the limbus, and place the implant on the same anatomical plane for all patients. The FIL-SSF was injected through the 2.2 mm corneal incision and the haptics were externalized with 25-gauge forceps through the sclerotomies. The haptics were placed under the flaps which were then sutured. The corneal incisions were closed with hydrosuture or a corneal suture when necessary (Fig. 1) [18].

#### Iris-claw ARTISAN® lens

The iris-claw lens (Artisan® aphakia model 205, Ophtec, Boca Raton, FL, USA) is rigid, made of polymethyl methacrylate (PMMA) with a 5.4 mm optic and 8.5 mm overall length. In this study the implant was inserted either through a 5.5 mm corneal incision or through a sclero-corneal tunnel aligned of the 12 o’clock meridian, using a three-step tunneled incision technique. Implant was positioned with the concave side facing up, and clipped to the posterior side of the iris at the 3 and 9 o’clock positions [25].

## Statistical analysis

Data were expressed as mean (standard deviation, SD) and count (percentage) for quantitative and qualitative variables, respectively. Quantitative variables were compared using Mann–Whitney U test, Welsh test or Student t-test. Qualitative variables were compared using Fisher or Chi2 test. All tests were two-tailed, and a *p*-value < 0.05 was considered as significant. Statistical analyses were performed using R software version 4.2.3 (R Foundation for Statistical Computing, Vienna, Austria).

## Results

### Patient’s characteristics

A total of 372 eyes of 363 patients were screened: 23 were excluded because the surgery was combined with keratoplasty, 21 were excluded because of corneal comorbidities, and 57 were excluded due to a lack of data, mainly because of the absence of reliable preoperative refractive data, or because patients were lost to follow-up. Finally, 271 (72.8%) eyes of 265 patients were included: 96 eyes in the FIL-SSF group, and 175 eyes in the iris-claw group. Baseline characteristics were well balanced between both groups (Table 1). The mean (SD) age was 70.3 (15.6) years in the FIL-SSF group and 70.5 (13.9) years in the iris-claw group (*p* = 0.70). Specifically, the different ocular biometry parameters were not significantly different according to surgery subgroup, including axial length (*p* = 0.69), preoperative sphere (*p* = 0,14), preoperative astigmatism (*p* = 0.61), and preoperative SE (*p* = 0.53). The preoperative mean (SD) BCVA was not significantly different in the FIL-SSF and the iris-claw group with respectively 0.70 (0.71) logMAR and 0.75 (0.72) logMAR. The main indications for secondary implantation were not significantly different between the two groups and included dislocated IOL (*p* = 0.19), followed by complicated cataract surgeries (*p* = 0.85). However, there were more patients implanted with FIL-SSF following ocular traumatisms (*p* = 0.03) or in case of uveitis glaucoma hyphema (UGH) syndrome (*p* = 0.001). The rate of secondary implantation combined with another surgical procedure (IOL explantation, epiretinal membrane peeling, silicon oil removal…) was not significantly different between the 2 groups (*p* = 0.85).

**Table 1:** Demographic and clinical characteristics of study eyes

	FIL-SSF group (*N* = 96)	Iris-claw group (*N* = 175)	*p*-value
Mean age, years (SD)	70.3 (15.6)	70.5 (13.9)	0.70
Gender, male, *n* (%)	68 (70.8)	110 (62.9)	0.19
Side, right, *n* (%)	45 (46.9)	88 (50.3)	0.65
Mean axial length, mm (SD)	24.2 (1.9)	24.2 (1.8)	0.69
Mean Preoperative astigmatism, diopters (SD)	1.45 (1.33)	1.45 (1.58)	0.61
Mean Preoperative sphere, diopters (SD)	6.36 (6.89)	5.05 (7.85)	0.14
Mean preoperative SE, diopters (SD)	5.6 (6.8)	5.0 (8.0)	0.53
Mean preoperative BCVA, logMAR (SD)	0.70 (0.71)	0.75 (0.72)	0.58
Mean IOP, mmHg (SD)	16.1 (6.7)	16.4 (16.2)	0.27
IOL Power, diopters (SD)	20.0 (4.8)	17.3 (4.2)	< 0.01
Preexisting comorbidities, *n* (%)	74.0 (77.0)	124.0 (70.9)	0.53
Surgical indication, *n* (%)			
IOL dislocation Complicated PKE surgery Traumatism UGH syndrome	43 (44.8) 28 (29.2) 11 (11.5) 10 (10.4)	93 (53.1) 53 (30.3) 8 (4.6) 2 (1.1)	0.19 0.85 0.03 0.001
Combined surgery, *n* (%)	28 (29.2)	53 (30.3)	0.85

*BCVA* Best Corrected Visual Acuity, *IOL* Intra Ocular Lens, *IOP* Intra Ocular Pressure, *PEX* Pseudo Exfoliation; *PKE* phacoemulsification, *SD* Standard Deviation, *SE* Spherical Equivalent, *UGH* Uveitis Glaucoma Hyphema

### Primary and secondary outcomes at 6 months

**Fig. 2:**
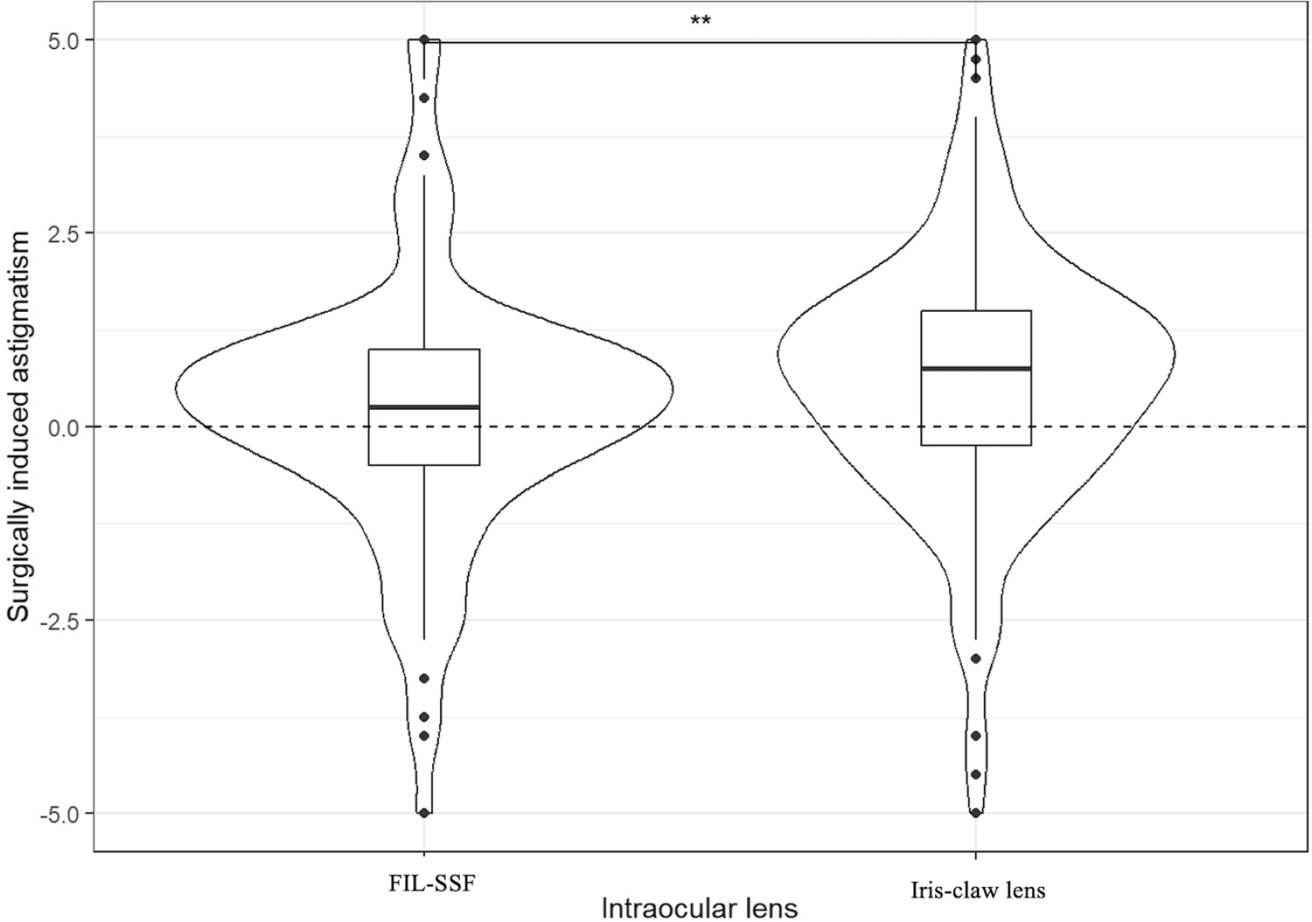
Hybrid violin plot and boxplot of surgically-induced astigmatism according to intraocular lens. For the boxplot, the bold line is the median of the distribution, hinges are 1st and 3rd quartile, whiskers are minimum and maximum and individual points are outliers. The difference is statically significant (*p* = 0.01)

The mean operating time was significantly different with longer mean (SD, [min–max]) surgery time for FIL-SSF procedure in comparison to iris-claw procedure with 59.8 (21.1, [23.0–164.0]) min versus 41.9 (24.4, [15.0–153.0]) min, respectively (*p* < 0.001). There was significantly more general anesthesia required for iris claw procedure in comparison with FIL-SSF (*p* = 0.02). Regarding the main outcome measure 6 months after the surgery, the mean (SD) SIA was significantly lower in the FIL-SSF group than in the iris-claw group (0.3 (1.8) diopters versus 0.8 (2.1) diopters, respectively, *p* = 0.01, Fig. 2). The mean postoperative sphere was significatively lower for the FIL-SSF group compared to the iris-claw group (0.3 (1.3) versus 0.8 (1.7) diopters, respectively (*p* < 0,001). Given a mean (SD) postoperative SE of -0.5 (1.4) for FIL-SSF and -0.01 (1.9) for iris-claw, the mean (SD) MRE was found significantly lower for the FIL-SSF group than in the iris-claw group (-0.1 (1.2) diopters versus 0.5 (1.6) diopters, respectively, *p* < 0.001). The postoperative mean BCVA was not significantly different between the two groups (*p* = 0.11, Table 2). In the subgroup of patients implanted with an iris-claw lens by sclero-corneal tunnel procedure (*n* = 25), the mean (SD) SIA was not significantly different than in the FIL-SSF group (0.4 (1.0) diopters versus 0.3 (1.8) diopters, respectively, *p* = 0.67).

**Table 2:** Comparison of main outcomes measures at 6 months after surgery

	FIL-SSF group (*N* = 96)	Iris-claw group (*N* = 175)	*p*-value
Mean operating time, min (SD)	59.8 (21.1)	41.9 (24.4)	< 0.001
General anesthesia, *n* (%)	25 (26.0)	71 (40.6)	0.02
Mean surgically induced astigmatism, diopters (SD)	0.3 (1.8)	0.8 (2.1)	0.01
Mean postoperative sphere, diopters (SD)	0.3 (1.3)	0.8 (1.7)	< 0.001
Mean refractive error, diopters (SD) Mean postoperative spherical equivalent, diopters (SD)	-0.1(1.2) -0.5 (1.4)	0.5 (1.6) -0.01 (1.9)	< 0.001
Mean BCVA, logMAR (SD)	0.5 (0.6)	0.4 (0.6)	0.11

*BCVA* Best Corrected Visual Acuity, *SD* Standard Deviation

### Complications

The mean (SD) follow-up was 9.3 (6.9) months for the FIL-SSF group and 11.5 (9.4) months for the iris-claw group. We reported 4 (4.1%) cases of peroperative complications in the FIL-SSF group and 2 (1.1%) cases in the iris-claw group. These included 2 (2.1%) cases of retinal detachment, 2 (2.1%) cases of haptic rupture in the FIL-SSF group, while 2 (1.1%) cases of suprachoroidal hemorrhage were reported in the iris-claw group.

Concerning postoperative complications, 30 (31.2%) eyes presented cystoid macular oedema in the FIL-SSF group compared to 41 eyes (23.4%) in the iris-claw group, but the difference was not statistically significant (*p* = 0.16). Haptic exposure was the main complication associated with FIL-SSF procedure (7.3% of cases) and all of them were noted within the first three months postoperatively. These patients required a surgical revision, autograft of conjunctival or scleral tissues, or amniotic membrane transplant. We noticed 3 (1.7%) cases of iris-claw lens dislocation, and 5 (2.9%) cases of postoperative hyphemia in the iris-claw group, while none were reported in the FIL-SSF group. Morevover, 8 (4.6%) cases of suprachoroidal detachment or hemorrhage were reported in the iris-claw group in comparison to 1 (1.0%) case in the FIL-SSF group. Other complications were rare and well-balanced between the 2 groups. There were no cases of endophthalmitis in either group (Table 3). There was 1 case of FIL-SSF IOL opacification in a patient treated for a rhegmatogenous retinal detachment 5 months after implantation. He was treated with cryotherapy and perfluoroethane (C2F6) gas tamponade which migrated in the anterior chamber, and implant opacification occurred 2 months after retinal detachment surgery.

**Table 3:** Postoperative complications during the whole follow-up

	FIL-SSF group (*N* = 96)	Iris-claw group (*N* = 175)
Mean follow up, months (SD)	9.3 (6.9)	11.5 (9.4)
Macular oedema, *n* (%)	30 (31.2)	41 (23.4)
Haptic exposure *n* (%)	7 (7.3)	–
Intra vitreous hemorrhage *n* (%)	4 (4.2)	6 (3.4)
Choroidal detachment or choroidal hematoma *n* (%)	1 (1.0)	8 (4.6)
Hyphema *n* (%)	0 (0)	5 (2.9)
IOL dislocation *n* (%)	0 (0)	3 (1.7)
Ocular hypertension *n* (%)	7 (7.3)	7 (4.1)
Ocular hypotony *n* (%)	0 (0)	1 (0.6)
Endophtalmitis *n* (%)	0 (0)	0 (0)
Endothelial decompensation *n* (%)	4 (4.2)	2 (1.1)
Retinal detachment *n* (%)	2 (2.1)	3 (1.7)
Postoperative Seidel *n* (%)	4 (4.2)	3 (1.7)

*IOL* Intra Ocular Lens, *IOP* Intra Ocular Pressure

## Discussion

The present study shows that FIL-SSF has a significantly lower SIA compared to the iris-claw implant. This result is most likely due to the smaller wound size and the absence of corneal sutures in almost all of the FIL-SSF implantations as it is performed through 2.2 mm incision, compared to a 6 mm sutured incision in the iris-claw group. A 6 mm wide corneal incision generally requires the placement of at least three corneal sutures and induces corneal deformation that can persist after suture removal. As expected, when the iris-claw is implanted through a sclero-corneal tunnel, the induced astigmatism is lower, and not significantly different than the FIL-SSF group. Other studies have also shown the advantage of sclero-corneal tunnel in minimizing induced astigmatism in iris-claw implantation [26]. However this technique is less commonly performed, possibly due to the fear of damaging the ciliary bodies and causing intraocular bleeding.

Regarding the mean refractive error, it was significantly lower in the FIL-SSF group, implying a better predictability of the postoperative refractive result. This may be partly because of the lower SIA, but also because of the greater stability of the FIL-SSF which is anchored directly to the sclera beneath flaps located 180° apart. Several studies comparing the FIL-SSF to the iris-claw lens have shown better results with the FIL-SSF, with a significantly lower SIA and MRE but on relatively small cohorts [22, 23]. The MRE of iris-claw implantation, in turn, has been estimated higher, probably due to the uncertainty of exact implant placement upon the iris.

In terms of postoperative BCVA, the difference between the two groups was not significant. As reported in the literature [22, 23], most patients had a better visual acuity after implantation, but in some cases here, it was limited by comorbidities such as ocular trauma or high myopia. As the rate of ocular complications is comparable, it is logical that visual acuity remains similar between the two implantations techniques. However, as FIL-SSF is a new implant, long term outcomes have yet to be described.

The duration of surgery was significantly longer for FIL-SSF with an operating time of nearly one hour, in comparison with the iris-claw implantation, which was around forty minutes. This is consistent with other studies, which found a surgery time averaging around one hour for FIL-SSF implantation [20, 21]. There is undoubtedly a steep learning curve for implantation of this new implant, as it is often performed by a retinal surgeon, and requires constructing scleral flaps, which is generally best performed by glaucoma surgeons. This translates into longer operating times for the first patients operated, which are included in this study, and for junior surgeons, less experienced in performing scleral flaps. However, once the learning curve is passed it should be noted here that the minimum operating time could be less than 30 min in experienced hands. In contrary, the surgery time for iris-claw implantation are found shorter than generally described in the literature, which report average times above one hour [23]. This can be explained herein by a longstanding experience in iris-claw implantation, as it is the preferred implant in aphakic eyes with no capsular support in our tertiary emergency centers. Also, it should not be forgotten that in both groups, the time of surgery could include other operating actions, such as IOL explantation, phakophagia, or silicon oil removal.

Regarding peroperative complications, the rate of haptic rupture during FIL-SSF externalization was low and similar to other studies [20, 27] and did not seem to compromise implant stability or require implant exchange. In fact, haptic rupture usually concerns only one end of the T-shape anchor, and the other end still provides enough support for correct scleral fixation.

Another complication associated with FIL-SSF implantation was retinal detachment, which occurred in 2 cases, and was probably due to the associated complete vitrectomy. On the contrary, iris-claw implantation generally does not require complete vitrectomy, and peroperative retinal detachment were not reported during the surgery, even though it can occur later during follow-up.

Concerning postoperative complications, the rate of adverse events was comparable to other studies, and there was no significant difference between the two groups. Interestingly, we noted around 30% of postoperative macular edema after FIL-SSF implantation which is more important than in other series which report rates between 5–7% [20, 27]. Frequency of postoperative macular edema was also higher for the iris-claw implantation comparatively to other series [12, 28]. This may be explained by a higher proportion of patients with other ophthalmological comorbidities or who had undergone multiple previous surgeries.

The second most common complication, which is specific to the FIL-SSF was scleral anchors erosion through the conjunctiva which occurred in about 7% of cases. Late-onset extrusion may be underestimated in our study as the follow-up is only 9 months. This is a serious complication that can lead to ocular infection, and its management is not standardized. All anchor extrusion cases in our series required additional surgery to properly cover the anchors, and a few cases relapsed. Other studies have shown smaller rates [27] and have identified thin flaps, thin sclera as seen in high myopia, ocular surface alterations and rheumatologic conditions with scleral inflammation as risk factors for erosion [29]. They subsequently recommended a flap thickness at least 60% of the sclera to limit this complication. They also propose a simple technique to move the implant into new pockets without removing it [29]. We suggest suturing the pockets to promote better healing of the sclera above the anchors. It is interesting to note that no case of FIL-SSF dislocation has ever been described, probably due to his particular design, which prevents the implant from moving secondarily. On the contrary, dislocation of the iris-claw lens is a relatively common complication [4, 28, 30], with weak or damaged iris. Endothelial cell counting was not systematically conducted in this study, but the rate of clinical endothelial decompensation was similar between the two groups and remained low. Other studies corroborate our observations and show only a moderate decrease in cell counting both for FIL-SSF and iris-claw implantation [19, 31].

Interestingly, we had one case of opacification of FIL-SSF opacification following gas tamponade, with anterior chamber gas migration one day after the surgery. There is seldom description of such cases in the literature [32]. One case of transient opacification has already been reported, attributed to rapid temperature change [33], and one case of permanent opacification was reported after DSAEK with multiple re-bubbling with air, and calcification on the anterior surface of the IOL [34]. While the main cause seems to be the presence of intracameral gas, other factors may include intraocular inflammation or diabetes mellitus [35].

This study has several limitations. Firstly, the retrospective design may account for incomplete data reporting and discontinuous follow-up. Secondly, FIL-SSF can be considered as a new technique, and a learning curve could drive results down for the first patients implanted. Moreover, the large experience of surgeon with iris-claw implantation could increase the outcomes with this implant. However, and despite these bias that could minimize our results, a significant superiority of FIL-SSF in terms of SIA and refractive error is seen in the present study.

In conclusion, FIL-SSF implantation might represent a useful surgical approach for the management of aphakia. Future studies not including the “learning curve”, including more patients in a prospective design could show a larger difference. Longer follow-up period is also needed to ascertain its tolerance.
